# The Impact of 12 h Night Shifts on Nurses’ Driving Safety

**DOI:** 10.3390/nursrep13010040

**Published:** 2023-03-08

**Authors:** Stephen Michael James, Lois James

**Affiliations:** 1 Elson S Floyd College of Medicine, Washington State University, Spokane, WA 99223, USA; 2 College of Nursing, Washington State University, Spokane, WA 99223, USA

**Keywords:** nurses, shiftwork, driving safety, fatigue, sleep restriction

## Abstract

***Aim***: The purpose of this study was to determine the impact of 12 h day vs. 12 h night shift-accumulated fatigue on nurses’ driving safety. ***Background***: Evidence across industries links work-related fatigue with errors, accidents, and adverse long-term health outcomes. Shifts of 12 h or longer are particularly problematic, and the potential risks to shift-worker driving safety during their post-shift commute home have yet to be fully explored. ***Methods***: This study used a between-groups, repeated-measures non-randomized control trial. Forty-four nurses working 12 h day shifts and 49 nurses working 12 h night shifts were tested in a driving simulator on two separate occasions—once immediately following their third consecutive 12 h hospital shift and once on their third consecutive day (72 h) off work. ***Results***: We found that night shift nurses had significantly greater lane deviation during the post-shift drive home compared to day shift nurses, which is a key indicator of collision risk, demonstrating impaired driving safety. ***Conclusions***: Consecutive 12 h night shifts are an extremely popular shift for nurses working in the hospital setting, however they pose a significant driving safety risk to nurses assigned to night shifts. This study provides objective evidence of the impact of shift work-related fatigue on 12 h night shift nurse safety, allowing us to make recommendations that may help prevent injury or death from motor vehicle collisions.

## 1. Background

Research indicates that shift-work-related fatigue is associated with safety, health, and performance impairments [1,2,3,4,5]. Twelve-hour shifts or longer appear to be particularly problematic, especially for night or rotating shift workers [6,7]. Despite these risks, 12 h shifts have become increasing popular for many shift-working professionals, including nurses [8]. In part, this is due to ease of scheduling within the hospital setting, and in part this is due to concerns over continuity of patient care suffering with shorter (e.g., 8 h) shifts. A major concern for nursing is that no national restrictions exist around work hours, meaning that a hospital could legally schedule a nurse for multiple back-to-back shifts. Although physician work hours tend to be longer than 12 h (e.g., 24 or even up to 48 h during residence), shift regulations have existed for them since 1987 due to the known risks of long work hours. In short, the health, safety, and performance of physicians has been a national consideration for decades, while nurses remain unprotected by national work-hour policy. 

Over the past decade or so the American Nursing Association (ANA) has emphasized the critical need to reduce nurse fatigue. This emphasis is fueled by research showing that shift-work-related sleep deprivation and chronic fatigue among nurses have been associated with increased risk of errors affecting quality of patient care [9,10]. In addition, research has connected nurse fatigue to occupational injury [11], burnout and compassion fatigue [12], and increased risk of disease in the long-term [13]. Fatigue has been recognized by the Joint Commission (a U.S. healthcare accrediting organization focused on enhancing patient safety and care) as a leading threat to both patient safety and healthcare worker safety [14].

Researchers have monitored shift workers during real world driving and found negative effects of night shift work in particular. For example, Ftouni and colleagues found that self-reported drowsy driving and adverse driving events were significantly higher following night shifts, and that this was particularly pronounced for workers who had been awake for 16 h or more [15]. Similarly, Lee and colleagues tested shift workers driving on a closed driving track and found that 37.5% of drives following a night shift resulted in a near collision compared to 0% of drives following a day shift [16]. Using self-reported measures to evaluate adverse outcomes on the drive home (post-shift), Anderson and colleagues found that physicians working extended duration work shifts were more than twice as likely to report adverse outcomes compared to their commute to work (pre-shift) [17]. Mulhall and colleagues focused specifically on nurses, asking them to keep a driving log recording any adverse events on their commutes to and from work. Like Anderson’s study, they found that participants reported more adverse driving events following night shifts, especially if they had been awake for more than 16 h [18]. 

In an attempt to better quantify the risks in a safe environment, driving simulation is increasingly being used to test the impact of shift work on driving safety. For example, Åkerstedt and colleagues demonstrated that night shift workers are at an increased risk of collision during simulated driving compared to day shift workers [19]. This same finding has been observed with police officers assigned to night shifts, who are at greater risk of collision during their “post-shift” drive home than day-shift officers when tested in a driving simulator [20]. Although the impact of shift work on nurse safety during the post-shift commute home has been investigated via self-reported driving logs, to date simulation studies of driving safety have not been conducted with nurse participants. 

In order to inform safe scheduling practices, quantitative estimates of the impact of 12 h day vs. 12 h night shifts on nurses’ safety risks while driving home are essential. Although prior studies have identified an increased risk of adverse events driving home for night shift workers, little is understood about the extent of those risks. In the midst of national demands to reduce the detrimental impact of fatigue on accidents and injuries, lawsuits against hospitals claiming nurses are being put at severe risk due to overwork, and the increased pressure of the COVID-19 pandemic on the nursing profession, research determining the impact of shift-accumulated fatigue on nurse safety is essential.

## 2. The Current Study

Testing the impact of three consecutive 12 h shifts on nurses’ driving safety was part of a larger between-groups, repeated-measures non-randomized control trial conducted in the Washington State University (WSU) College of Nursing and Sleep and Performance Research Center (SPRC) to determine the impact of shift work on nurse and patient safety. This manuscript describes results related to nurse safety; patient safety results have been previously reported [21]. Three consecutive shifts were chosen as that was the typical work schedule for nurses in the hospital we recruited from. As part of this experiment, 44 nurses working 12 h day shifts and 49 nurses working 12 h night shifts were tested in a high-fidelity driving simulator on two separate occasions—once following their third consecutive 12 h shift and once on their third consecutive day (72 h) off work. Conditions were counterbalanced among participants to control for learning effects. Throughout the course of the experiment, participants were tested on 90 min of critical skills in a nursing simulation lab to estimate patient care skills [21], and a 20 min drive in a driving simulator to simulate a post-shift drive home (results reported here). 

The specific research question addressed here is whether nurses are at increased risk of a driving collision following 12 h night shifts compared to 12 h day shifts. Our main research hypothesis was that night shift nurses would be at greater risk for a driving collision than day shift nurses.

## 3. Methods

### 3.1. Study Design

We employed a mixed repeated-measures (post-shift vs. time off), between-groups (12 h day vs. 12 h night shift) design. Participants (N = 94) came to the lab on two occasions—once immediately after their third consecutive 12 h shift (“on-duty” condition), and once at the same time of day on their third consecutive day off (“off-duty” condition). Order of testing was randomized to avoid learning effects. Testing occurred at the same time of day for each condition to control for circadian factors. Shift patterns at the hospital nurses were recruited from were 07:00–19:00 for days and 19:00–07:00 for nights. Thus, day-shift nurses arrived at the lab at approximately 19:30 on both testing days and were tested in the driving simulator at approximately 21:00, while night-shift nurses arrived at the lab at approximately 07:30 on both testing days and were tested in the driving simulator at approximately 09:00. 

Our rationale for testing nurses immediately after their third consecutive shift was because this was frequently their final shift in a work week, so arguably when they had the most shift-accumulated fatigue that they were likely to have. Similarly, our rationale for testing nurses again on their third consecutive day off was that this was their penultimate day off, resulting in them being rested but still allowing them time to recover (post-testing) so they could get sufficient sleep before returning to work. 

### 3.2. Sample and Setting

This study was approved by the WSU Institutional Review Board (IRB) prior to participant recruitment (IRB# 16739-006). In total 50 nurses working the 12 h day shift and 50 nurses working the 12 h night shift were recruited from a local medical center (sample calculated from a power analysis based on a previous study of ours testing police participants). Due to the COVID-19 pandemic, data collection concluded slightly earlier than intended, resulting in a final sample of 94 participants. Given that this represented over 90% of our target enrolment, we opted to end the study instead of pause until data collection was feasible again. 

Our recruitment plan was coordinated with the medical center, and included posting flyers, posting on hospital websites and social media sites, announcements at staff meetings, and word-of-mouth via research coordinators. To encourage nurses to volunteer, we paid overtime rates for study consenting at the hospital and the full amount of time between getting off shift and completing testing in our lab on two separate occasions. This resulted in approximately 6 h in total of reimbursed time. 

Furthermore, to ensure nurse safety, on “work” condition testing days we arranged Uber rides for participants for the duration of the day. This included a trip from home to work, from work to the lab, and from the lab back home. This was necessary to avoid placing participants at increased risk for collisions following what amounted to a 14.5-h shift (their regular 12 h shift in the hospital, travel time to the lab, and approximately 2 h of testing at the lab). 

**Figure 1:**
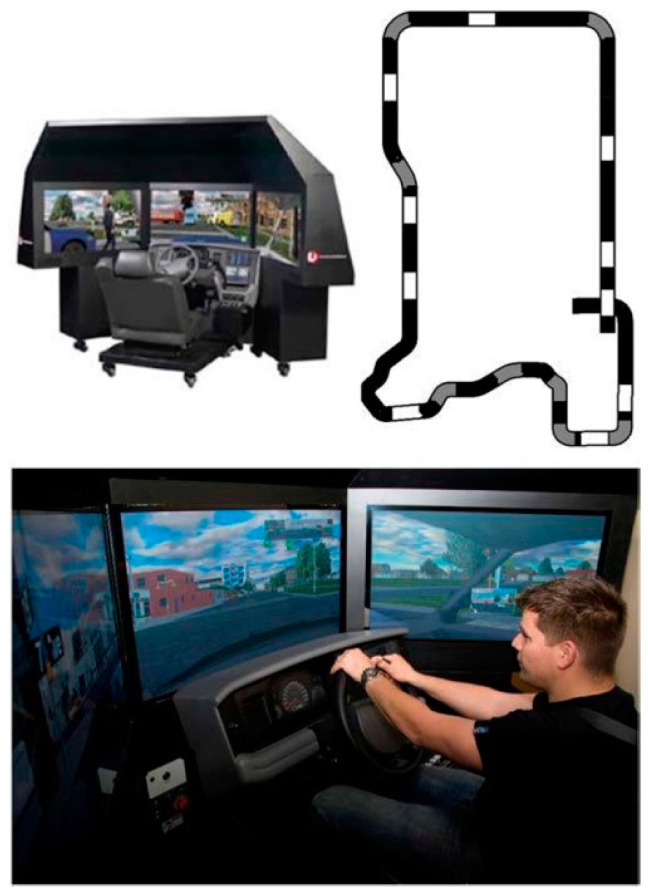
MPRI PatrolSim IV driving training simulator (top left), structure of the 28-mile road course with measured straights in white and measured curves in gray (top right), and a demonstration of driving the simulator in a suburban scenario rather than the rural highway scenario used in these experiments.

The driving portion of testing occurred In the SPRC, which includes HD driving simulators (see Figure 1), in a light- and sound-controlled environment, with custom developed driving metrics for quantifying risk of collision. The SPRC is a coalition of basic and applied research laboratories aiming to understand the neurobiology of sleep and sleep loss, and the effect of sleep loss on metabolism, immune function, cognitive performance, and behavior, all with the aim of ensuring adequate, recuperative sleep and/or mitigating the effects of inadequate sleep.

### 3.3. Study Procedures

As nurses volunteered to participate, we screened for eligibility. This included hospital level “fit-to-work” clearance, as well as requiring that all participants were “healthy sleepers.” Specifically, during screening we excluded participants with known sleep disorders such as insomnia or sleep apnea, or participants that were not cleared to work in the hospital. We anticipated that this would result in a more conservative estimate of the impact of shift work on risks to nurses and their patients, and also reduce the risk of selection bias (e.g., nurses volunteering because they wanted to gain insight into their poor sleep). 

All screening was conducted via telephone by the study coordinator, following a screening protocol. When eligible participants were identified, they were randomly assigned to either on-duty condition first, or off-duty condition first, and were scheduled for testing days. At least 4 weeks were allotted between testing days to try and reduce study learning effects. 

Towards the end of the testing session, participants completed a 20 min drive in a driving simulator. During testing participants were closely monitored by research assistants, under the guidance of the study coordinator and the research team. At the conclusion of testing, participants were debriefed, and either scheduled for their second session or discharged from the study if all testing was complete. 

## 4. Measures

The SPRC is equipped with two high-fidelity MPRI PatrolSim IV driving training simulators that collect all the commonly measured parameters used in simulator-based driving research. The simulators capture data on variables such as speed, lane-keeping, frequency of braking, steering control, accelerator release time, time to collision, accelerator-to-braking transition time, and minimum acceleration. Each of these is thought to be affected by driver fatigue. Data are sampled at 72 Hz. The scenario used in this study was a 20 min drive in a rural setting. During the scenario, participants are randomly presented with unexpected events such as a pedestrian or dog running in front of their vehicle, the driver ahead braking suddenly, or a speeding vehicle roaring past. Performance during this drive is based on embedded metrics for driving consistency (e.g., lane deviation, braking latency, and collisions).

### 4.1. Data Management

Data were stored on a secure server at WSU. To guard against loss, all data were backed up regularly. Scanned study forms were archived with the PI and stored in a secure location. All study data were de-identified by assigning each participant a unique study identification number (SID) that was included on all study forms’ data files. This SID was used to link multiple forms completed by the participant. A crosswalk database was maintained that linked the SID to each participant’s name and other identifying information required. This database was password protected and kept in a secure location with restricted access. De-identified data were merged in Excel via SID and imported to SPSS for analysis.

### 4.2. Study Hypotheses and Analytical Approach

Our main research hypothesis was that night shift nurses would be at greater risk for driving collision than day shift nurses. Additional research hypotheses were that nurses would be at greater risk during their on-duty condition compared to their off-duty condition, and that an interaction effect would exist whereby night shift nurses during their on-duty condition would be at the greatest risk for collision. To estimate the risk of collisions in the driving simulator, we measured known collision predictors: lane deviation, braking latency (time to brake), and braking violence, which is a measure of the force applied to braking (from 0% to 100% braking), divided by the amount of time the brake is depressed until maximum braking is reached (time in seconds), a higher value representing an abrupt or violent braking action in contrast to a lower value representing a more gradual or steady braking event. We also measured actual collisions in the driving simulator, although we anticipated that there would be too few for meaningful analysis. 

We used multi-level-modeling (MLM)—with adjustments for multiple comparisons—to analyze the data. This analytical technique accounts for clustering of observations around participants, and partitions variance accordingly, reducing the risk of type I errors by avoiding violating the assumption of independence among observations. It was used as an alternative to repeated measures Analysis of Variance (ANOVA), which has greater difficulty coping with missing data than MLM. IBM SPSS (v. 24.0.0.0, New York, NY, USA) was used for all statistical analysis.

## 5. Results

Ninety-four nurses participated in the study, out of a target enrolment of 100 (this number was established by an a priori power analysis). Table 1 describes the sample characteristics and demographics.

**Figure 2:**
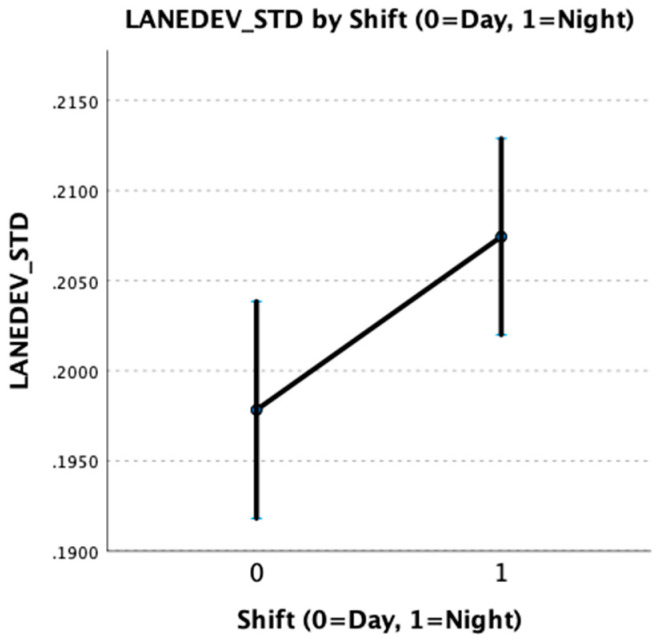
Standardized lane deviation (LANEDEV_STD) by participant work shift.

Multi-level models revealed that participants working the 12 h night shift had significantly greater lane deviation than participants working the 12 h day shift (f = 5.40; df = 1, 1588; *p* < 0.05). Our primary research hypothesis was therefore supported, indicating that night shift nurses were at greater risk for collision than day shift nurses. This is illustrated in Figure 2 below, with error bars indicating 95% confidence intervals.

Testing of additional research hypotheses revealed that although participants had a trend of greater lane deviation during their on-duty condition than during their off-duty condition, this did not reach significance at the 0.05 alpha level (f = 2.45, df = 11,588; *p* = 0.12). 

**Figure 3:**
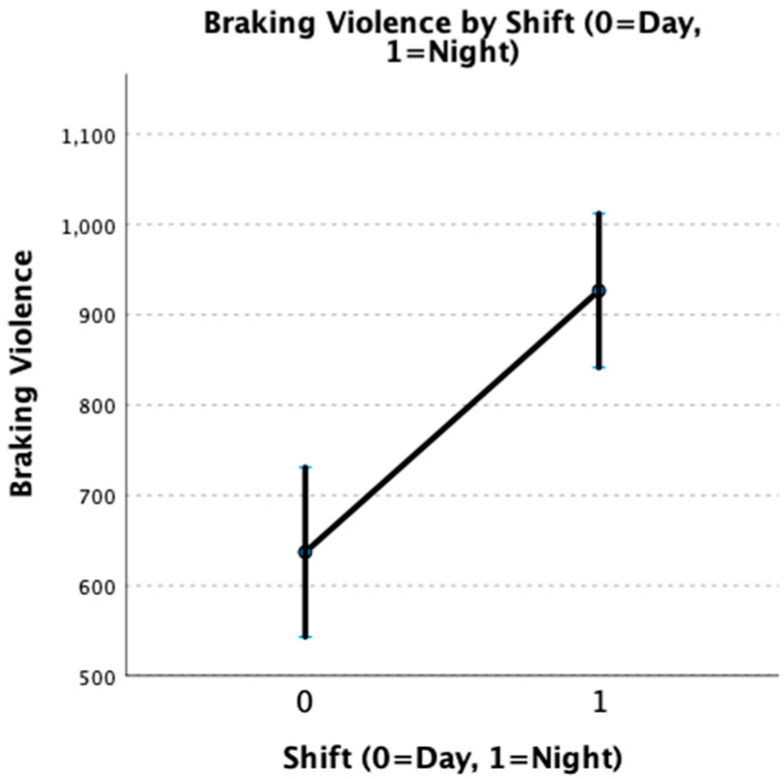
Braking violence by participant work shift.

Although no significant differences in braking latency based on either shift (day vs. night) or condition (on-duty vs. off-duty) were observed, night shift nurses had significantly greater braking violence (f = 20.12, df = 1, 840; *p* < 0.001). This indicates that night shift nurses had a greater startle response in the simulator than day shift nurses, regardless of whether they were on- or off-duty. More abrupt and violent braking may lead to increased risk of rear-end collision or losing control of their vehicle during braking. This is illustrated in Figure 3 below. 

**Figure 4:**
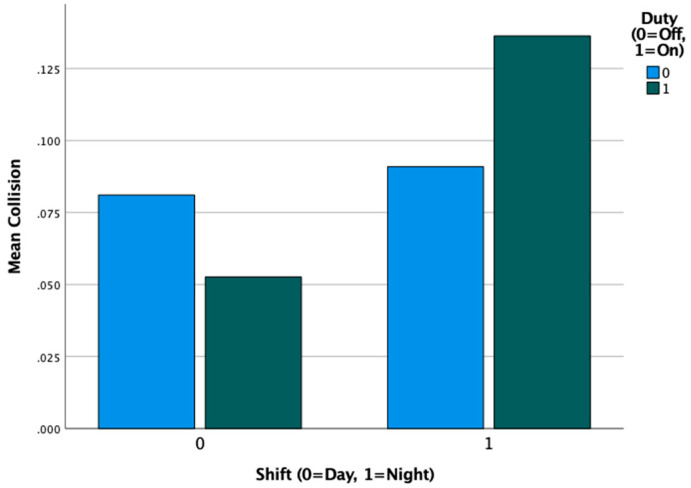
Simulator collisions by shift type (day vs. night) and shift duty (on vs. off).

When looking at collisions in the simulator, we found that 10% of participants had a collision during their on-duty condition compared to 9% during their off-duty condition, which was not a statistically significant difference. A larger discrepancy was observed for day vs. night shift participants, with day shift participants experiencing collisions in 7% of drives, compared to night shift participants who had collisions in 11% of drives. This difference did not quite meet the 0.05 alpha threshold of significance, so we urge caution in interpreting this result. Figure 4 illustrates that the group most at-risk for collision was the night shift group during their on-duty test session.

No significant interactions between shift type (day vs. night) and shift duty (on-duty vs. off-duty) were observed for any outcome variables, indicating that night shift nurses were at greater risk for collision, even following days off work. 

## 6. Discussion

Regarding nurse safety, our study findings reveal that night shift participants had significantly greater lane deviation than day shift participants, which is a key indicator of collision risk, demonstrating impaired driving safety. These findings add to the growing body of evidence across industries linking night-shift-related fatigue with accidents and adverse outcomes. In addition, they support national healthcare organization concerns about sleep restriction, fatigue, and waking sleepiness as hazards for healthcare workers’ safety. Of particular note in this study—night shift nurses were at increased risk of collision compared to day shift nurses, even after three consecutive days off. 

Our findings are in alignment with previous research on the impact of night shifts, specifically on nurse safety. For example, Smart and Wilson found that night shift nurses report more drowsiness driving home after shifts than day shift nurses, which aligns with our finding of increased lane deviation and risk of collision [22]. Similarly, Ftouni and colleagues found that night shift nurses reported increased drowsy driving and adverse driving events compared to day shift nurses, especially when they were sleep deprived (in this case defined as being awake for 16 h or more) [15]. 

In the hospital setting, 12 h shifts are popular due to ease of scheduling, as well as some nurses’ preferences for consolidated time off-duty, and beliefs about continuity of patient care. Given, however, the established risks associated with 12 h night shifts, interventions must be considered to promote patient and healthcare worker safety alike. These could include education about sleep hygiene (optimizing sleep environment to maximize sleep opportunity), training on fatigue countermeasures (e.g., light therapy, caffeine, exercise, nutrition), and sleep or fatigue monitoring. Additionally, policies at the hospital level could help decrease safety risks for nurses and patients alike, such as introducing on-duty napping, providing rideshare opportunities, or matching nurses chronotype (how much of a morning or an evening person they are) to shifts in order to decrease work/sleep dyssynchronization. 

Several study limitations need to be addressed that may influence the generalizability of results. Although simulation is a valuable tool for use as a proxy for real world skills, it is still an artificial environment, without some of the inherent risks and stresses of real-world performance. That said, in situations where real measures are unsafe (for example driving) we argue that simulation provides the most realistic and valid alternate measure. We also argue that simulation allows for a much more precise estimate of risks compared to self-reported measures such as driving logs. Another limitation is the possibility of selection bias on the part of the nurses who chose to volunteer. 

## 7. Conclusions

Our study findings are directly relevant to current occupational and environmental health nursing practice in that they provide objective quantifiable evidence of the safety risks to nurses driving home following 12 h night shifts. This information adds to the body of evidence justifying the need for policies and practices designed to reduce the risks of fatigue and promote nurse safety. Given that 12 h shifts are unlikely to disappear, the probable solution is to reduce on-the-job fatigue, promote nurses’ sleep health, and consider workplace policies such as on-duty napping which could significantly alleviate safety risks. Additional policies and practices that could reasonably be applied to nurses include putting a barrier in place to remove one of the critical safety risks associated with 12 h shifts: post-shift driving safety. Hospitals should consider providing lifts home to nurses, especially those most at risk for collision, which our findings indicate are night shift nurses.

## Figures and Tables

**Table 1:** Sample Characteristics by Shift Type.

	Night Shift (*n* = 49)	Day Shift (*n* = 44)	All (*n* = 93)
Age (*SD*)	34.14 (9.14)	37.96 (9.59)	35.93 (9.52)
Female (%)	87.76	95.18	89.36
Non-White * (%)	14.29	9.09	11.70
Bachelor’s Degree (%)	69.39	77.27	73.40
Patient Focus (%)			
Neonatal	20.41	13.64	17.02
Pediatric	30.61	40.91	35.11
Adult/Geriatric	48.98	45.45	47.87

*Note*. *SD* = standard deviation. All variables were determined by self-report. * Non-White (%) includes Hispanic-White.
